# Are Diverse Media Better than a Single Medium? The Relationship between Mixed Media and Perceived Effect from the Perspective of Online Psychological Counseling

**DOI:** 10.3390/ijerph18168603

**Published:** 2021-08-14

**Authors:** Jingfang Liu, Lu Gao

**Affiliations:** School of Management, Shanghai University, Shanghai 201800, China; jingfangliu2014@hotmail.com

**Keywords:** media richness, mixed media, electronic health, perceived effect, social support, satisfaction

## Abstract

The progress of new media has promoted the development of online health consultations. Previous research has investigated the impact of media richness on user satisfaction; however, little attention has been given to the mixed effects of the nesting of multiple media. The purpose of this study is to analyze the impact and differences of the use of single or mixed media on users’ perceived effect from the perspectives of social support and satisfaction by mining user reviews on online health platforms. The data were collected from a professional online psychological counseling platform. We collected data on 48,807 reviews from 11,694 users. Text annotation and sentiment analysis were then used to extract variable eigenvalues from the reviews. One-way analysis of variance (ANOVA) and hierarchical regression analysis were used for statistical analysis. The results show that mixed media with different richness has a significant impact on the users’ perceived effects. Among them, compared to “text + audio,” using “text + audio + video/face to face” can significantly improve the users’ perceived social support and satisfaction. However, compared to single medium, mixed media with higher richness may not necessarily achieve a better effect. We found that the inclusion of “video/face to face” mixed media significantly reduced the users’ perceived social support and satisfaction compared to text or audio use alone. These research results complement the blank media richness theory in the field of online health care and provide guidance for improving the personalized customization of online psychological counseling platforms.

## 1. Introduction

### 1.1. Background

With the development of the Internet and the popularization of new media, Internet-based health consultation has been booming. An increasing number of users are seeking doctors and exchanging health information through network platforms [1]. Currently, online consultation has become the main way for users to seek health help. Compared to traditional offline outpatient services, users tend to think that online medical services can bring more convenience and choice [2].

The forms of doctor–patient communication on Internet health platforms have evolved over time. Early online consultation was mainly text-based real-time chat in which patients communicated health information with doctors by sending text or images [3]. In the process of text communication, limited content and a lack of timeliness were gradually revealed [4]. Therefore, as a medium with high timeliness, wide transmission, and strong communication, audio has been widely introduced [5]. To further enhance the social presence of online consultations, many doctors have recently introduced video features. The addition of visual information provides a new understanding of online health development and has attracted extensive research attention [6,7].

Although online communication media have attracted much attention from scholars in different fields, most of the literature has focused on factors that affect user choices (timeliness, multichannel communication prompts, language diversity, etc.) [8,9] and perceived effects (trust, communication efficiency, satisfaction, etc.) [10,11] in the case of a single medium. Few studies have focused on the users’ perceived effects under the mixed use of media with different richness. Moreover, from the perspective of media richness theory in the information systems research field, user output performance and continuous use behavior in online learning communities [8] and game communities dominate the research [12]. However, there is still a large research gap in the attitudes and perceptions of users in online health communities [13].

Under the development trends of refinement, customization, and individualization, information systems pay more attention to targeted research on specific populations and markets [14,15]. In the field of public health, mental health is an important topic [16]. On the one hand, mental illness represented by depression and insomnia can lead to very severe consequences, such as suicidal behavior [17,18]. On the other hand, compared to the treatment of physiological diseases such as cancer and liver disease through drugs, most mental illnesses can be improved and eradicated through professional consultation [19,20,21]. According to a definition by Barak et al., psychological counseling is an interactive process based on specific technical media (email, audio, video, forums, etc.) to establish contact between professional counselors and clients [22]. Therefore, patients with mental illness have higher requirements for the matching and interactivity of communication media when using network-based health services [23]. Finally, from the perspective of mental health caregivers, the use of technical media can help select and reduce admissions to a psychiatric urgent care service by circumscribing access to the most serious cases [20,24,25,26,27]. Based on the above reasons, we chose online mental health consultation as our research context.

In the past, the literature on the media behavior of patients with mental illnesses was mainly divided into two research directions. One is the study of user media selection and output performance under a single medium [28,29,30]. This type of research uses interviews to collect data. For example, Paul’s research pointed out that audio media has obvious advantages in quickly responding to patient needs [31]; Liu et al.’s research showed that text consultation is suitable for the expression of more complex emotions [32]. The other main research direction does not consider specific media attributes or analyze the impact of media richness on the users’ perceived value on the macro level [10,33,34]. Instead, this type of research uses questionnaires to collect data. For example, scholars such as Jorge distributed questionnaires in online communities, exploring the relationship between mobile technology and the perceived social support of depression patients [35]. According to the above literature review, we found that the current application of media richness theory in online psychological counseling lacks research on the mixed effects of media nesting. Regarding the influence of media on performance, most previous studies used cross-sectional data and lacked dynamic data and the continuous tracking of user media behavior.

Online user reviews contain a wealth of information that can help us understand user needs, emotional attitudes, and value perception [36,37,38]. At the same time, each review corresponds to a point in time and can reflect the changing process of the users’ mental states over a period of time, which is of great significance when studying user dynamic behavior choices and changes in perceived effects. For example, using online reviews, Ju et al. analyzed the factors affecting the continuous use of online health treatments by diabetic patients [39]. The research in this paper collects media selection and reviews feedback of users using online psychological counseling over a period of time and uses text analysis methods to determine perceived social support and emotional satisfaction from the review content as two factors to measure the perceived effect.

### 1.2. Research Question

Based on the current reality and theoretical background, this article uses the online mental health consulting community as the research scenario based on the content from online user reviews from the perspectives of social support and satisfaction. The article studies the impact and difference of using single or mixed media on the users’ perceived effects. There are two problems that are addressed:

Question 1: In network-based mental health consultation, how does the different richness of mixed media affect the user perceived effect?

Question 2: In network-based mental health consultation, what is the difference between the impact of a single medium or mixed media on the users’ perceived effects?

The research in this article provides new perspectives and new ideas for the research of media richness theory in the field of health. At the same time, it provides guidance for improving user recommendations on online psychological consulting platforms and personalized customized services.

## 2. Materials and Methods

### 2.1. Study Aim and Design

This research is based on the user review content from an online psychological consultation platform. Using the effective data of 11,694 users after screening, we analyzed the impact of using different richnesses of a single medium or mixed media on the user perceived effects, specifically from the perception of social support and satisfaction.

The research of this article is divided into two parts. In the first part, we conducted research on 4954 users who use mixed media. One-way analysis of variance (ANOVA) explores the influence of different richnesses of mixed media on the users’ perceived social support and satisfaction as well as the comparative relationship between different media combinations. In the second part, we examine the data of 6740 users using a single medium. Using text and audio media as benchmarks, we analyze the changes on the impact of user perceived social support and satisfaction after adding richer media.

### 2.2. Data Collection and Processing

The data of this study come from a professional Internet psychological counseling platform. The platform provides counseling services in four media (text, audio, video, and face-to-face) for psychological counseling clients. After the user accepts the psychological consultation, he can review and provide feedback on the personal page of the selected expert.

We collected all user reviews from 448 male consultants and 1437 female consultants from 2016 to early June 2021. After the preliminary deletion of duplicate values, we obtained 180,871 valid reviews. Specifically, we collected valid data tags, including reviewer avatar URL, reviewer nickname, review content, review time, and consultation method. For user privacy protection, the platform does not disclose user-related personal information or the specific process and content of the consultation. Therefore, our research is a combination of qualitative and quantitative analysis based on online reviews from the user perspective. This method of using online reviews to mine user opinions and attitudes has been proven to be feasible and effective [39,40].

The specific process of data processing is described as follows: We first classify users through two data tags, avatar URL, and nickname. This method of processing ensures that the users we screen are unique. Considering that the focus of this research is on mixed media usage behaviors under dynamic panel data, we then deleted users with only one review dataset among these users that were initially screened out. Finally, we sorted each review data of each user in the order of time from front to back to obtain the dynamic data of each user over a period of time. After a series of classifications and summary processing, we retained 48,807 valid review data points containing 11,694 independent users.

### 2.3. Variable Definition and Measurement

The variables involved in the research are sorted in Table 1. We choose the user perceived social support and satisfaction as indicators to measure the perceived effects and considered them separately. The specific variable measurement process is as follows:

#### 2.3.1. Social Support

Social support refers to the help and support that people receive from all aspects of social networks [41]. Previous studies showed that perceived social support improves user trust [42] and well-being [10] in online communities and enhances the willingness to continue to use them [43]. Therefore, we choose information support and emotional support in social support as dependent variables to measure the user perceived effects. Information support refers to the content of the consultants providing professional knowledge, advice, and guidance in the service reflected in the review text; emotional support refers to the content of the consultants supporting the emotional expression of the client, providing empathy or companionship via the service reflected in the review text.

With reference Lu et al.’s text analysis method of online depression community user posts [44], we use keyword extraction to mark user reviews and to establish ordered categorical variables: “1” represents information support, “2” represents emotional support, and “3” represents both information and emotional support. Considering that each user has more than two review data points, in the subsequent statistical analysis, the result of the mean calculation is used as a measure of each user’s perceived social support. Note that psychological counseling is aimed at people with psychological discomfort or psychological obstacles with the help of professional psychological methods of counselors to achieve the effect of restoring psychological balance, improving adaptability, physical health, and mental health [45]. That is, in psychological counseling, the transfer of professional knowledge and the use of specific methods ultimately help clients to obtain better emotional counseling services. Therefore, when we perform orderly marking, the value of “emotional support” is higher than that of “information support.”

#### 2.3.2. Satisfaction

Satisfaction refers to users’ psychological feelings and subjective evaluation of the quality of a relationship and is an important indicator that measures service quality and perceived effect [46]. For example, research by scholars such as Elena showed that patient satisfaction has a positive effect on strengthening the trust between doctors and patients and increasing the loyalty of using online health consultations [47]. Therefore, we take satisfaction as the second important factor to measure the perceived effect.

Since most of the user ratings in this article are 5 stars, it lacks reference value. According to the research experience of previous scholars, there is often a large difference between the real emotion of the score and the content of the review [36]. Therefore, we measure user satisfaction by calculating the sentiment score of the text from the user reviews. This article adopts the sentiment dictionary method. First, we look for emotional words in the emotional vocabulary, use each emotional word as a benchmark to find adverbs of degree, and multiply different weights according to the types of adverbs of degree to calculate the corresponding scores. The scores of each emotional word in the clause are then summed. Finally, the sentiment score is processed to prevent negative numbers from appearing [48]. Similar to perceived social support, in the subsequent statistical analysis, the result of the mean calculation is used as a measure of each user’s satisfaction.

#### 2.3.3. Media Types and Mixed Media

Mixed media is defined relative to a single medium, which refers to the nested use of media with different richness. Media richness can be divided into different types according to the timeliness of feedback, channel scalability, and clue diversity [49]. On the basis of human research and the development of today’s media, according to the order of media richness from low to high [50], four types of mixed media with different richness are divided (Table 1). Among them, “low” (L) represents text consultation media (text/image), “medium” (M) represents audio consultation media (voice), and “high” (H) represents video or face-to-face consultation media (voice + vision).

### 2.4. Statistics

This article used two statistical analysis methods for two different research questions: one-way analysis of variance (ANOVA) and hierarchical regression analysis. The specific reasons and handling details of the selected methods are as follows:

#### 2.4.1. One-way Analysis of Variance (ANOVA)

**Figure 1:**
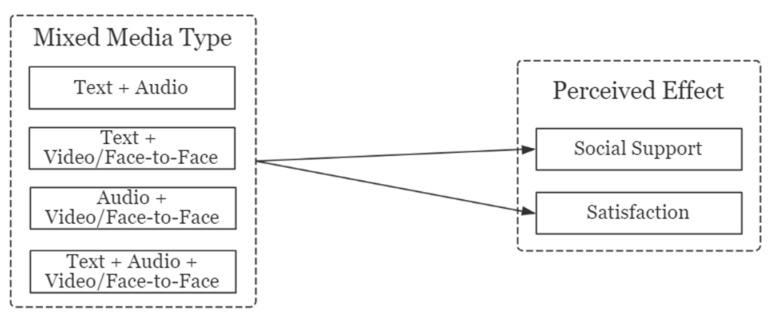
Research model 1.

In the first part, our research aim is to verify whether mixed media with different richness will affect the users’ perceived effects. Therefore, we divided four different types of mixed media, and analyzed the impact on perceived social support and satisfaction separately. Figure 1 illustrates the research model of the first part.

The main principle of ANOVA is to test the significance of the difference in the mean of the dependent variable when only one factor is changing. Before using ANOVA for research, two important assumptions need to be met: (a) the dependent variable within each group must conform to the normal distribution, and (b) satisfy the condition of the homogeneity of variance. We used Shapiro–Wilk for the normality test. The results in Table 2 and Table 3 show that our data do not conform to the normal distribution (*p* < 0.05). The significance of the Levene variance homogeneity test is greater than 0.05 (Table 4), and it can be considered that the variance between the sample data is homogeneous. In this case, we did not choose the non-parametric test (Kruskal–Wallis test) to replace the ANOVA, mainly for two reasons: On the one hand, the studies of McDonald and many other scholars have shown that the results of ANOVA (parametric test) are not sensitive to the situation where normality is not satisfied [51,52], so normality conditions cannot be considered too much in practical applications. On the other hand, for large samples, benefiting from the central limit theorem, even if the data is not normal, the sample mean is always approximately normal [53]. Therefore, under the condition that the data sample size is large enough and the homogeneity of variance is satisfied, we ignore the unsatisfied factors of normality and still adopt ANOVA.

ANOVA includes three steps: First, perform a simple descriptive statistical analysis of the relevant variables and initially observe the differences in the mean of each group. Then, run the analysis of variance to observe whether the results show a significant difference (*p*-value) and use the effect size to study the magnitude of the difference in-depth (partial η^2^). Finally, we conduct a post-test to further clarify which groups are different if the results of the analysis of variance are significant. Because our data meet the homogeneity of variance and because the sample size of each group is different, we chose the Scheffe method for post hoc comparison [54] and analyzed the significance of the mean difference at the level of α = 0.05.

Using this method, it is not only possible to compare whether there is a difference in the mean values of the perceived social support and satisfaction under four different types of mixed modes, but to also judge whether there is a difference in the difference between before and after changes.

#### 2.4.2. Hierarchical Regression Analysis

In the second part, our research aim is to compare the difference between the impact of single medium and mixed media on the user perceived effect. We selected four different types of media: two single media (“text” and “audio”) and two mixed media (“text + video/face-to-face” and “audio + video/face-to-face”) because the greater the difference in media richness, the easier it is to compare the difference in results.

**Figure 2:**
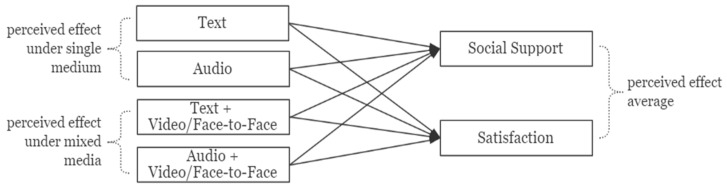
Research model 2.

Figure 2 illustrates the research model of the second part. The dependent variables are the average value of the perceived effect, which we calculated based on all the reviews of each user. The independent variables are the users’ perceived effect under the four different types of media that we have chosen. Perceived effects are calculated from the two perspectives of social support and satisfaction separately. Additionally, for each type of media that the user has not chosen, we uniformly mark the perceived social support and satisfaction as “0” (in the previous data processing, the two variables have been processed to prevent negative numbers and “0” from appearing).

We use the hierarchical regression method for verification. By gradually adding new variables on the basis of the previous variables, hierarchical regression can reflect the importance of each independent variable to the dependent variable (*p*-value) as well as the change of the prediction model (R^2^). In the process of hierarchical regression, first, we analyzed the impact of two single media (text and audio) on user perceived effect; then, we added two types of mixed media (“text + video/face-to-face” and “audio + video/face-to-face”), observing the changes in the model’s interpretation ability; finally, we further added a single medium (audio) and mixed media (audio + video/face-to-face) interaction terms, analyzing the significance level of the independent variables and the explanatory power of the final model. In order to avoid the multicollinearity problem from interfering with the research results of this article, we calculated the VIF value for verification and explanation.

## 3. Results

### 3.1. One-Way Analysis of Variance (ANOVA)

#### 3.1.1. Impact of Mixed Media on Perceived Social Support

The descriptive statistics of perceived social support are listed in Table 5. We ran a one-way analysis of variance (ANOVA) on perceived social support. The results (Table 6) show that the use of mixed media with different richness has a significant impact on users’ perception of social support (F (3,4950) = 3.73, *p* < 0.05, η^2^ = 0.002). Furthermore, we made three comparisons of the four types of mixed media (as shown in Table 7). The results show that compared to the mixed media of “text + audio,” using “text + audio + video/face-to-face” can significantly improve users’ perceived social support (*p* < 0.05).

#### 3.1.2. Impact of Mixed Media on Satisfaction

The descriptive statistics of satisfaction are shown in Table 8. We ran a one-way analysis of variance (ANOVA) on satisfaction. The results (Table 9) show that mixed media with different richness has a significant impact on user satisfaction (F (3,4950) = 5.97, *p* < 0.001, η^2^ = 0.004). Furthermore, we made three comparisons of the four types of mixed media (as shown in Table 10). The results show that compared to the mixed media of “text + audio” and “audio + video/face-to-face,” using “text + audio +video/face-to-face” can significantly improve user satisfaction (*p* < 0.01). In addition, the difference in satisfaction between “text + audio + video/face-to-face” and “audio + video/face-to-face” is higher than that of “text + audio.”

Overall, the results of the ANOVA show that for psychological counseling users who use mixed media, nested forms of media with different richness have a significant impact on users’ perceived social support and satisfaction. However, the difference is small. The effect on satisfaction (F (3,4950) = 5.97, *p* < 0.001, η^2^ = 0.004) is little higher than that of perceived social support (F (3,4950) = 3.73, *p* < 0.05, η^2^ = 0.002). In addition, under the media mix of “text + audio + video/face-to-face,” users’ perceived social support and satisfaction were significantly improved.

### 3.2. Hierarchical Regression Analysis

We use partial least squares (PLS) for multiple regression analysis. PLS can effectively solve the problem of multicollinearity in independent variables. Specifically, we analyzed the differences in user perceived effect under different rich media from two single media (“text” and “audio”) and two mixed media (“text + video/face-to-face” and “audio + video/face-to-face”). The descriptive statistical results of each variable are shown in Table 11. In the actual process, due to the multicollinearity between some variables, we obtained the regression results shown in Table 12 through analysis. In addition, the calculation results of VIF are shown in Table 13.

The results of Table 12 show that in Model 1 and Model 4, the perceived social support and satisfaction of text and audio both positively and significantly affect the average perceived effect during the entire consultation process. Among them, the perceived social support (β = 0.413, *p* < 0.001) and satisfaction (β = 0.461, *p* < 0.001) with audio media have more obvious influences.

Model 2 and Model 5 added video/face-to-face on the basis of text and audio, respectively. The results show that ➀ the user perceived effect of “text + video/face-to-face” has a significant negative correlation with the average perceived social support (β = −0.094, *p* < 0.001) but does not correlate with the average satisfaction. ➁ The user perceived effect of “audio + video/face-to-face” is significantly positively correlated with average perceived social support (β = 0.055, *p* < 0.001) and is significantly negatively correlated with average satisfaction (β = −0.034, *p* < 0.01).

The results of Model 3 and Model 6 reveal that there is a significant interaction between a single “audio” medium and a mixed media of “audio + video/face-to-face.” Among them, the perceived social support of the audio medium weakened the positive impact of the users’ perceived social support under the mixed media of “audio + video/face-to-face” on the average perceived social support (β = −0.035, *p* < 0.001). The satisfaction of “audio” media enhanced the negative impact of user satisfaction under the mixed media of “audio + video/face-to-face” on average satisfaction (β = 0.218, *p* < 0.001).

We calculated the VIF value to determine whether there is a multicollinearity problem between variables. Table 13 shows that all VIF values are below 5. Therefore, in the research model of this article, the problem of multicollinearity will not become an interference factor that affects the research results.

## 4. Discussion

### 4.1. Principal Findings

From the perspectives of social support and satisfaction, this paper studies the influence and the difference of the use of single and mixed media on the users’ perceived effect in the process of online psychological counseling. The main findings are as follows:

(1) Mixed media with different richness levels have a significant impact on the users’ perceived effects. Among them, the “text + audio + video/face-to-face” media nesting method can significantly improve users’ perceived social support and satisfaction to the greatest extent.

First, let us consider the personal characteristics of users. They are willing to try different rich media to communicate, which shows that the users themselves have a better acceptance of network media, the skills needed to use it, and running-in effects. Second, considering the characteristics of the media, the form of “text + audio + video/face-to-face” reached the highest degree in terms of media richness and media diversity. Users can freely switch between different media communication methods to meet their current needs without being restricted by objective conditions such as time and location, thereby avoiding the shortcomings of a single medium. In addition, the cross-mixed use of “text,” “voice,” and “vision” can enhance the breadth and depth of information and emotional transmission, and in a gradual manner, the two parties can increase mutual trust and intimacy in a continuous running-in. Ultimately, the effect of improving user perceived social support and satisfaction is achieved.

(2) Compared to a single medium, mixed media with higher richness does not necessarily obtain a better perceived effect. In this study, in addition to the mixed media of “audio + video/face-to-face,” which can improve user perceived social support, a mixture of text or audio and video/face-to-face media significantly reduces user perceived social support and satisfaction. Regarding these two novel findings, the following possible explanations are presented:

a. According to media interaction theory [57], different media forms ultimately affect the communication effect by changing the pace, scale, and content of the interaction process. The communication rhythm of “text” media is relatively slow, and the amount of information carried is moderate but is suitable for the stage of preliminary understanding and running-in between the two parties. “Video/face-to-face” communication is a formal form of communication when both parties have reached a high level of familiarity and trust. In psychology, the “gradual effect” refers to the natural tendency of people to maintain cognition and behavioral consistency with others in their subconsciousness. The direct conversion from “communication in front of the screen” to “face-to-face” does not conform to the normal development sequence of human-to-human communication. To a large extent, it enhances the sense of abruptness between the two parties, breaks the originally established emotional connection, and reduces the users’ perceived effects in the psychological consultation process.

b. The theory of perceptual interactivity states that [58] the users’ control of the communication rhythm plays an important role in shaping the perception of interactivity. Since audio communication cannot see the true situation of both parties, users can express their personal emotions in a more relaxed manner, and it is then easier to grasp the dominant power in the communication process and enhance the interaction. The addition of “visual” information reduces the users’ control over the surrounding environment and the things in it. However, the newly generated information elements cause user unfamiliarity and discomfort; this situation requires a certain amount of running-in time. Therefore, users’ perceived interactivity in the communication process is reduced, which in turn weakens the users’ overall satisfaction. However, face-to-face conversation increases the counselor’s ability to capture the client’s body and movement information, thereby improving the accuracy of problem judgment and enhancing the information and emotional support perceived by the user.

(3) “Audio” media can significantly adjust the impact of “audio + video/face-to-face” on the user perceived effect. Specifically, the positive impact of “audio + video/face-to-face” on perceived social support is weakened with the enhancement of users’ perceived social support under “audio” media. The negative impact of “audio + video/face-to-face” on satisfaction increases with the enhancement of user satisfaction under “audio” media.

According to the results of the descriptive statistics and multiple regression, it is not difficult to find that the users’ perceived effects under “audio” media has the most significant positive impact on the users’ average perceived effect of the entire consultation process. At the same time, users rely the most on audio media for consultation. This situation is largely because users’ trust and security perception of audio media are higher than those of video/face-to-face media. Audio communication puts users in a comfortable communication zone. On the one hand, they can obtain timely and effective professional psychological guidance. On the other hand, they can better protect their privacy on the basis of ensuring sufficient information transmission. Therefore, users show a clear preference for a “medium” rich audio in media selection, which in turn forms an important regulatory role for audio in the mixing of different media.

### 4.2. Theoretical Contribution and Practical Significance

The theoretical contributions of this research are as follows:

(1) It expanded the theory of media richness. The current literature based on media richness theory has mostly focused on the selection and influence of a single medium. Few scholars have paid attention to user selection and the perceived effects of the use of mixed media with different richness. Second, research on media and output effects have mostly used cross-sectional data, and few documents have continued to track user media behavior and perception changes within a certain period of time. The research in this article is an important supplement to research on the dynamic behavior of users under mixed media.

(2) This study compared the effects of three different richness media: text, audio, and video/face-to-face. To the best of our knowledge, there has been almost no research comparing the three different media at the same time or on how their mixed forms affect the perceived social support and satisfaction of online psychological counseling. This research is of great significance for understanding user media selection and perceived effects based on online psychological counseling.

(3) This research provides a new direction for the future use of online review research and the expansion of media richness theory. The research results of this paper show that through the content of user reviews on online platforms, the relationship between media selection and user behavior perception can be explored. Most of the predecessors used questionnaire surveys and group interviews to conduct research, which largely limited the scope of the population and the amount of data. This research is a novel attempt to use secondhand data for research, which opens up a new perspective on media richness research.

The practical significance of this research is as follows:

(1) The research of this article has two practical implications. On the one hand, this research provides meaningful guidance to help mental health workers to detect the needs of patients in a timely and effective manner and to overcome the barriers of telemedicine technology [59,60]. We analyzed the content of reviews generated online by users and could fully and accurately understand the direct feedback and counseling suggestions of psychological counseling patients, which is of great help for improving and perfecting the service quality of psychological counselors.

(2) On the other hand, this research provides some guidance for the communication between counselors and clients in network-based psychological counseling services [16,25]. The results of this study show that “text + audio +video/face-to-face” mixed media can maximize the perceived effects of clients. However, both parties should pay attention to the fact that despite greater media richness and more diverse forms, the best perceived effect might not be produced in the final consultation result. It is necessary to carefully consider factors such as the specific form of the media combination, user usage habits, and the degree of time run-in. When one is unsure about which combination of media will produce better results, the “audio” medium can be used as a priority.

### 4.3. Limitations

This study has three main limitations that may affect the generality and comprehensiveness of the research results. First, in terms of data collection, since people could choose on their own whether to write a user review or not, some user review data may be lost. Moreover, the use of video and face-to-face video is less frequent, and further research is needed, which can be achieved by expanding data sources to confirm and expand the validity and universality of the results. The second limitation, which is also limited by research data, is that we cannot obtain information about user characteristics such as gender and income. Adding these user attributes is of great significance to the comprehensiveness of the research. Finally, the research goal of this article was to focus on the influence of mixed media on the users’ perceived effects. We did not study the antecedents that affect the users’ choice of mixed forms of media; this also provides a direction for future research.

## 5. Conclusions

From the perspective of online mental health consultation and based on the theory of media richness, this paper studied the impact of a single medium and mixed media on the users’ perceived effects. The results show that mixed media with different richness levels have a significant impact on the users’ perceived social support and satisfaction. However, compared to a single medium, mixed media with higher richness does not necessarily obtain a better effect. The use of a single “text” or “audio” medium has a more significant positive impact on the users’ perceived effects than mixed media that includes “video/face-to-face.” This article pointed out a number of meaningful directions for further study of the impact of perceived effects of online health platform users from the perspective of media richness.

## Figures and Tables

**Table 1:** Operationalization of variables.

Node Attribute	Type	Measuring Method
Social support	Categorical variable and Continuous variable	1-Information support; 2-Emotional support; 3-Information & Emotional support. Mean value is used as a dependent variable indicator.
Satisfaction	Continuous variable	Sentiment score calculated based on user review text.
		Mean value is used as a dependent variable indicator.
Single Medium	Categorical variables	1- Text: Contains text or image information
		2- Audio: Contains voice information
		3- Video/Face-to-Face: Contains voice and visual information
Mixed Media ^1^	Categorical variables	1- L + M: Text + Audio
		2- L + H: Text + Video/Face-to-Face
		3- M + H: Audio + Video/Face-to-Face
		4- L + M + H: Text + Audio + Video/Face-to-Face

Notes: ^1^ “L” (low) represents the lowest rich medium—text media; “M” (medium) represents middle rich medium—audio media; “H” (high) represents the highest rich medium—video or face-to-face media.

**Table 2:** Tests of normality—perceived social support in mixed media.

Group	Shapiro–Wilk		
	Statistic	df	Sig.
1 Text + Audio	0.935	3467	0.000
2 Text + Video/Face-to-Face	0.929	40	0.015
3 Audio + Video/Face-to-Face	0.925	545	0.000
4 Text + Audio + Video/Face-to-Face	0.890	902	0.000

Notes: Significance: *p* > 0.05, indicating that the data conforms to the normal distribution.

**Table 3:** Tests of normality—satisfaction in mixed media.

Group	Shapiro–Wilk		
	Statistic	df	Sig.
1 Text + Audio	0.818	3467	0.000
2 Text + Video/Face-to-Face	0.951	40	0.085
3 Audio + Video/Face-to-Face	0.947	545	0.000
4 Text + Audio + Video/Face-to-Face	0.931	902	0.000

Notes: Significance: *p* > 0.05, indicating that the data conforms to the normal distribution.

**Table 4:** Test for homogeneity of variances—perceived social support and satisfaction.

	Levene Statistics	df1	df2	Sig.
Perceived social support	0.859	3	4950	0.462
Satisfaction	1.676	3	4950	0.170

Notes: Significance: *p* > 0.05, indicating that the variances of each group are equal.

**Table 5:** Descriptive statistics—perceived social support in mixed media.

Mixed Media	*n*	Mean	SD	Min	Max
1 Text + Audio	3467	2.090	0.550	1	3
2 Text + Video/Face-to-Face	40	2.162	0.550	1	3
3 Audio + Video/Face-to-Face	545	2.101	0.578	1	3
4 Text + Audio + Video/Face-to-Face	902	2.159	0.565	1	3
Total	4954	2.105	0.557	1	3

Notes: *n*: sample size; SD: standard deviation.

**Table 6:** Results of ANOVA—perceived social support in mixed media.

Source	df	Mean Square	F	Sig.	Partial η^2^
Between groups	3	1.153	3.73	0.011 *	0.002
Within groups	4950	0.309			
Total	4953				

Notes: Significance: * *p* < 0.05. η^2^ effect size: 0.1, 0.25, and 0.40 indicate small, medium, or large predictive relevance [55].

**Table 7:** Multiple comparisons of mixed media on perceived social support.

Group (I)	Group (J)	Mean Difference (I–J)	Sig.	95% Confidence Intervals	
1	2	−0.071	0.885	−0.319	0.176
	3	−0.010	0.984	−0.082	0.061
	4	−0.068	0.013 *	−0.126	−0.010
2	3	0.061	0.930	−0.194	0.316
	4	0.003	1.000	−0.248	0.254
3	4	−0.058	0.298	−0.142	0.027

Notes: Significance: * *p* < 0.05.

**Table 8:** Descriptive statistics—satisfaction in mixed media.

Mixed Media	*n*	Mean	SD	Min	Max
1 Text + Audio	3467	9.125	0.867	6	28.833
2 Text + Video/Face-to-Face	40	9.109	0.608	8	10.240
3 Audio + Video/Face-to-Face	545	9.094	0.735	7	12.600
4 Text + Audio + Video/Face-to-Face	902	9.248	0.747	7.678	12.917
Total	4954	9.144	0.832	6	28.833

Notes: *n*: sample size; SD: standard deviation.

**Table 9:** Results of ANOVA—satisfaction in mixed media.

Source	df	Mean Square	F	Sig.	Partial η^2^
Between groups	3	4.117	5.97	0.000 ***	0.004
Within groups	4950	0.690			
Total	4953				

Notes: Significance: *** *p* < 0.001. η^2^ effect size: 0.1, 0.25, and 0.40 indicate small, medium, or large predictive relevance [55].

**Table 10:** Multiple comparisons of mixed media on satisfaction.

Group (I)	Group (J)	Mean Difference (I–J)	Sig.	95% Confidence Intervals	
1	2	−0.071	1.000	−0.354	0.385
	3	−0.010	0.887	−0.076	0.138
	4	−0.068	0.001 ***	−0.210	−0.036
2	3	0.061	1.000	−0.366	0.396
	4	0.003	0.786	−0.514	0.237
3	4	−0.058	0.009 **	−0.280	−0.027

Notes: Significance: *** *p* < 0.001, ** *p* < 0.01.

**Table 11:** Descriptive statistics—perceived social support and satisfaction.

VARIABLE	*n*	Mean	SD	Min	Max
Social support (AVE)	11,694	2.111	0.538	1	3
Text	11,694	0.941	1.104	0	3
Audio	11,694	1.929	0.734	0	3
Text + Video/Face-to-Face	11,694	0.169	0.579	0	3
Audio + Video/Face-to-Face	11,694	0.278	0.718	0	3
Satisfaction (AVE)	11,694	9.163	1.235	4.500	86.33
Text	11,694	4.130	4.620	0	24.40
Audio	11,694	8.367	2.880	0	86.33
Text + Video/Face-to-Face	11,694	0.744	2.524	0	13
Audio + Video/Face-to-Face	11,694	1.135	3.037	0	13.33

Notes: *n*: sample size; SD: standard deviation. This part of the research data includes 4954 users who used mixed media and 6740 users who used a single medium. Therefore, for the types of media that have not been used, we marked the corresponding perceived social support and satisfaction as “0” (we have processed “satisfaction” as non-negative and non-zero before). Therefore, the descriptive statistical results in this part are clearly distinguished from Table 5; Table 8.

**Table 12:** Regression result—perceived social support and satisfaction.

VARIABLE	Social Support					Satisfaction				
	Model 1	Model 2	Model 3	95% Confidence Intervals		Model 4	Model 5	Model 6	95% Confidence Intervals	
Text	0.191 ***	0.216 ***	0.221 ***	0.099	0.116	0.145 ***	0.158 ***	0.150 ***	0.035	0.045
Audio	0.413 ***	0.419 ***	0.413 ***	0.290	0.315	0.461 ***	0.469 ***	0.595 ***	0.246	0.265
Text + Video/Face-to-Face		−0.094 ***	−0.100 ***	−0.118	−0.068		−0.021	−0.027 *	−0.026	−0.001
Audio + Video/Face-to-Face		0.055 ***	0.075 ***	0.035	0.077		−0.034 **	−0.155 ***	−0.075	−0.051
Audio × (Audio + Video/Face-to-Face)			−0.035 ***	−0.071	−0.020			0.218 ***	0.047	0.060
R^2^	0.177	0.180	0.181			0.203	0.205	0.223		
*n*	11,694	11,694	11,694			11,694	11,694	11,694		

Notes: Significance: *** *p* < 0.001, ** *p* < 0.01, * *p* < 0.05. R^2^ values: 0.25, 0.50, and 0.75 indicate weak, moderate, and substantial predictive power [56]. The regression coefficients shown in Table 12 are standardized coefficients. At the same time, we added a 95% confidence interval to the regression coefficients of Model 3 and Model 6. In order to eliminate multicollinearity, we centralized the interaction items. The processing method is to subtract the average value from each variable.

**Table 13:** Multicollinearity test—perceived social support and satisfaction.

VARIABLE	VIF					
	Social Support			Satisfaction		
Text	1.039	1.212	1.240	1.056	1.237	1.240
Audio	1.039	1.058	1.094	1.056	1.083	1.954
Text + Video/Face-to-Face		2.641	2.688		2.653	2.656
Audio + Video/Face-to-Face		2.398	2.868		2.418	3.216
Audio × (Audio + Video/Face-to-Face)			1.357			2.587

## References

[1] Wu T., Deng Z., Chen Z., Zhang D., Wang R., Wu X. (2019). Predictors of Patients’ Intention to Interact With Doctors in Web-Based Health Communities in China: Cross-Sectional Study. J. Med. Internet Res..

[2] Wu T., Deng Z., Chen Z., Zhang D., Wu X., Wang R. (2019). Predictors of Patients’ Loyalty Toward Doctors on Web-Based Health Communities: Cross-Sectional Study. J. Med. Internet Res..

[3] Paul C.L., Cox M.E., Small H.J., Boyes A.W., O’Brien L., Rose S.K., Baker A.L., Henskens F.A., Kirkwood H.N., Roach D.M. (2017). Techniques for Improving Communication of Emotional Content in Text-Only Web-Based Therapeutic Communications: Systematic Review. JMIR Ment. Health.

[4] Navarro P., Sheffield J., Edirippulige S., Bambling M. (2020). Exploring Mental Health Professionals’ Perspectives of Text-Based Online Counseling Effectiveness With Young People: Mixed Methods Pilot Study. JMIR Ment. Health.

[5] Rigotti N.A., Tindle H.A., Regan S., Levy D.E., Chang Y., Carpenter K.M., Park E.R., Kelley J.H., Streck J.M., Reid Z.Z. (2016). A Post-Discharge Smoking-Cessation Intervention for Hospital Patients: Helping Hand 2 Randomized Clinical Trial. Am. J. Prev. Med..

[6] Funderskov K.F., Raunkiaer M., Danbjorg D.B., Zwisler A.D., Munk L., Jess M., Dieperink K.B. (2019). Experiences With Video Consultations in Specialized Palliative Home-Care: Qualitative Study of Patient and Relative Perspectives. J. Med. Internet Res..

[7] Byaruhanga J., Atorkey P., McLaughlin M., Brown A., Byrnes E., Paul C., Wiggers J., Tzelepis F. (2020). Effectiveness of Individual Real-Time Video Counseling on Smoking, Nutrition, Alcohol, Physical Activity, and Obesity Health Risks: Systematic Review. J. Med. Internet Res..

[8] dos Santos H.L., Cechinel C. (2018). The final year project supervision in online distance learning: Assessing students and faculty perceptions about communication tools. Behav. Inf. Technol..

[9] van Houwelingen C.T., Ettema R.G., Antonietti M.G., Kort H.S. (2018). Understanding Older People’s Readiness for Receiving Telehealth: Mixed-Method Study. J. Med. Internet Res..

[10] Chan M., Li X. (2020). Smartphones and psychological well-being in China: Examining direct and indirect relationships through social support and relationship satisfaction. Telemat. Inform..

[11] Paredes M.R., Apaolaza V., Fernandez-Robin C., Hartmann P., Yanez-Martinez D. (2021). The impact of the COVID-19 pandemic on subjective mental well-being: The interplay of perceived threat, future anxiety and resilience. Pers. Individ. Dif..

[12] Liao G.-Y., Huang T.-L., Cheng T.C.E., Teng C.-I. (2020). Impacts of media richness on network features and community commitment in online games. Ind. Manag. Data Syst..

[13] Gieselmann A., Podleschka C., Rozental A., Pietrowsky R. (2021). Communication Formats and Their Impact on Patient Perception and Working Mechanisms: A Mixed-Methods Study of Chat-Based vs. Face-to-Face Psychotherapy for Insomnia. Behav. Ther..

[14] Kujala S., Ammenwerth E., Kolanen H., Ervast M. (2020). Applying and Extending the FITT Framework to Identify the Challenges and Opportunities of Successful eHealth Services for Patient Self-Management: Qualitative Interview Study. J. Med. Internet Res..

[15] Toscos T., Coupe A., Flanagan M., Drouin M., Carpenter M., Reining L., Roebuck A., Mirro M.J. (2019). Teens Using Screens for Help: Impact of Suicidal Ideation, Anxiety, and Depression Levels on Youth Preferences for Telemental Health Resources. JMIR Ment. Health.

[16] Shore J.H., Hilty D.M., Yellowlees P. (2007). Emergency management guidelines for telepsychiatry. Gen. Hosp. Psychiatry.

[17] Lin Y.H., Chiang T.W., Lin Y.L. (2020). Increased Internet Searches for Insomnia as an Indicator of Global Mental Health During the COVID-19 Pandemic: Multinational Longitudinal Study. J. Med. Internet Res..

[18] Costanza A., Ambrosetti J., Wyss K., Bondolfi G., Sarasin F., Khan R. (2018). Prévenir le suicide aux urgences : De la « Théorie Interpersonnelle du Suicide » à la connectedness [Prevention of suicide at Emergency Room: From the « Interpersonal Theory of Suicide » to the connectedness]. Rev. Med. Suisse.

[19] Liu J., Kong J., Zhang X. (2020). Study on Differences between Patients with Physiological and Psychological Diseases in Online Health Communities: Topic Analysis and Sentiment Analysis. Int. J. Environ. Res. Public Health.

[20] Butterfield A. (2018). Telepsychiatric Evaluation and Consultation in Emergency Care Settings. Child Adolesc. Psychiatr. Clin. N. Am..

[21] Seidel R.W., Kilgus M.D. (2014). Agreement between telepsychiatry assessment and face-to-face assessment for Emergency Department psychiatry patients. J. Telemed. Telecare.

[22] Barak A., Klein B., Proudfoot J.G. (2009). Defining internet-supported therapeutic interventions. Ann. Behav. Med..

[23] Mirzaei T., Kashian N. (2020). Revisiting Effective Communication Between Patients and Physicians: Cross-Sectional Questionnaire Study Comparing Text-Based Electronic Versus Face-to-Face Communication. J. Med. Internet Res..

[24] Cowan K.E., McKean A.J., Gentry M.T., Hilty D.M. (2019). Barriers to Use of Telepsychiatry: Clinicians as Gatekeepers. Mayo Clin. Proc..

[25] Salmoiraghi A., Hussain S. (2015). A Systematic Review of the Use of Telepsychiatry in Acute Settings. J. Psychiatr. Pract..

[26] Narasimhan M., Druss B.G., Hockenberry J.M., Royer J., Weiss P., Glick G., Marcus S.C., Magill J. (2015). Impact of a Telepsychiatry Program at Emergency Departments Statewide on the Quality, Utilization, and Costs of Mental Health Services. Psychiatr. Serv..

[27] Costanza A., Mazzola V., Radomska M., Amerio A., Aguglia A., Prada P., Bondolfi G., Sarasin F., Ambrosetti J. (2020). Who Consult an Adult Psychiatric Emergency Department? Pertinence of Admissions and Opportunities for Telepsychiatry. Medicina.

[28] Bird M.D., Chow G.M., Meir G., Freeman J. (2019). The Influence of Stigma on College Students’ Attitudes Toward Online Video Counseling and Face-to-Face Counseling. J. Coll. Couns..

[29] Bleyel C., Hoffmann M., Wensing M., Hartmann M., Friederich H.C., Haun M.W. (2020). Patients’ Perspective on Mental Health Specialist Video Consultations in Primary Care: Qualitative Preimplementation Study of Anticipated Benefits and Barriers. J. Med. Internet Res..

[30] Cipolletta S., Mocellin D. (2018). Online counseling: An exploratory survey of Italian psychologists’ attitudes towards new ways of interaction. Psychother. Res..

[31] Paul C.L., Boyes A.W., O’Brien L., Baker A.L., Henskens F.A., Roos I., Clinton-McHarg T., Bellamy D., Colburn G., Rose S. (2016). Protocol for a Randomized Controlled Trial of Proactive Web-Based Versus Telephone-Based Information and Support: Can Electronic Platforms Deliver Effective Care for Lung Cancer Patients?. JMIR Res. Protoc..

[32] Liu J., Gao L. (2021). Analysis of topics and characteristics of user reviews on different online psychological counseling methods. Int. J. Med. Inform..

[33] Mirzaei T., Esmaeilzadeh P. (2021). Engagement in online health communities: Channel expansion and social exchanges. Inf. Manag..

[34] Zhang Z., Zhang L., Xiao H., Zheng J. (2021). Information quality, media richness, and negative coping: A daily research during the COVID-19 pandemic. Personal. Individ. Differ..

[35] Arias-de la Torre J., Puigdomenech E., Garcia X., Valderas J.M., Eiroa-Orosa F.J., Fernandez-Villa T., Molina A.J., Martin V., Serrano-Blanco A., Alonso J. (2020). Relationship Between Depression and the Use of Mobile Technologies and Social Media Among Adolescents: Umbrella Review. J. Med. Internet Res..

[36] Lee S.-W., Jiang G., Kong H.-Y., Liu C. (2020). A difference of multimedia consumer’s rating and review through sentiment analysis. Multimed. Tools Appl..

[37] Sun X., Han M., Feng J. (2019). Helpfulness of online reviews: Examining review informativeness and classification thresholds by search products and experience products. Decis. Support Syst..

[38] Chen M.-J., Farn C.-K. (2020). Examining the Influence of Emotional Expressions in Online Consumer Reviews on Perceived Helpfulness. Inf. Process. Manag..

[39] Ju C., Zhang S. (2020). Influencing Factors of Continuous Use of Web-Based Diagnosis and Treatment by Patients With Diabetes: Model Development and Data Analysis. J. Med. Internet Res..

[40] Guo Y., Barnes S.J., Jia Q. (2017). Mining meaning from online ratings and reviews: Tourist satisfaction analysis using latent dirichlet allocation. Tour. Manag..

[41] Renfrew M.E., Morton D.P., Morton J.K., Hinze J.S., Przybylko G., Craig B.A. (2020). The Influence of Three Modes of Human Support on Attrition and Adherence to a Web- and Mobile App-Based Mental Health Promotion Intervention in a Nonclinical Cohort: Randomized Comparative Study. J. Med. Internet Res..

[42] Peng Y., Yin P., Deng Z., Wang R. (2019). Patient-Physician Interaction and Trust in Online Health Community: The Role of Perceived Usefulness of Health Information and Services. Int. J. Environ. Res. Public Health.

[43] Wu T., Deng Z., Feng Z., Gaskin D.J., Zhang D., Wang R. (2018). The Effect of Doctor-Consumer Interaction on Social Media on Consumers’ Health Behaviors: Cross-Sectional Study. J. Med. Internet Res..

[44] Lu Y., Luo S., Liu X. (2021). Development of Social Support Networks by Patients With Depression Through Online Health Communities: Social Network Analysis. JMIR Med. Inform..

[45] Pretorius C., Chambers D., Cowan B., Coyle D. (2019). Young People Seeking Help Online for Mental Health: Cross-Sectional Survey Study. JMIR Ment. Health.

[46] Deng Z., Lu Y., Wei K.K., Zhang J. (2010). Understanding customer satisfaction and loyalty: An empirical study of mobile instant messages in China. Int. J. Inf. Manag..

[47] Platonova E.A., Kennedy K.N., Shewchuk R.M. (2008). Understanding patient satisfaction, trust, and loyalty to primary care physicians. Med. Care Res. Rev..

[48] Xie R., Chu S.K.W., Chiu D.K.W., Wang Y. (2021). Exploring Public Response to COVID-19 on Weibo with LDA Topic Modeling and Sentiment Analysis. Data Inf. Manag..

[49] Lengel R.H., Daft R.L. (1983). Information richness: A new approach to managerial behavior and organizational design. Res. Organ. Behav..

[50] Lee Y., Kozar K.A., Larsen K.R. (2009). Avatar e-mail versus traditional e-mail: Perceptual difference and media selection difference. Decis. Support Syst..

[51] Lix L.M., Keselman J.C., Keselman H.J. (1996). Consequences of Assumption Violations Revisited: A Quantitative Review of Alternatives to the One-Way Analysis of Variance “F” Test. Rev. Educ. Res..

[52] McDonald J.H. (2014). Handbook of Biological Statistics.

[53] Troncoso Skidmore S., Thompson B. (2013). Bias and precision of some classical ANOVA effect sizes when assumptions are violated. Behav. Res. Methods.

[54] Lee S., Lee D.K. (2018). What is the proper way to apply the multiple comparison test?. Korean J. Anesthesiol..

[55] Chin W.W. (1998). The partial least squares approach for structural equation modeling. Mod. Methods Bus. Res..

[56] Henseler J., Ringle C.M., Sinkovics R.R. (2009). The use of partial least squares path modeling in international marketing. Adv. Int. Mark..

[57] Sundar S.S., Jia H., Waddell T.F., Huang Y. (2015). Toward a Theory of Interactive Media Effects (TIME). The Handbook of the Psychology of Communication Technology.

[58] McMillan S.J., Hwang J.-S. (2013). Measures of Perceived Interactivity: An Exploration of the Role of Direction of Communication, User Control, and Time in Shaping Perceptions of Interactivity. J. Advert..

[59] Deslich S., Bruce S., Tomblin S., Coustasse A. (2013). Telepsychiatry in the 21st Century: Transforming Healthcare with Technology. Perspect. Health Inf. Manag..

[60] Bhugra D., Tasman A., Pathare S., Priebe S., Smith S., Torous J., Arbuckle M.R., Langford A., Alarcon R.D., Chiu H.F.K. (2017). The WPA-Lancet Psychiatry Commission on the Future of Psychiatry. Lancet Psychiatry.

